# Supplementary material for: High Incidence of SARS-CoV-2 Variant of Concern Breakthrough Infections Despite Residual Humoral and Cellular Immunity Induced by BNT162b2 Vaccination in Healthcare Workers: A Long-Term Follow-Up Study in Belgium
Source: Viruses. 2022 Jun 9;14(6):1257. doi: 10.3390/v14061257 (PMC9228150; doi:10.3390/v14061257)
Supplement: Supplementary file 1 [file viruses-14-01257-s001.zip › viruses-1742727-supplementary.pdf]

viruses

Supplementary Materials

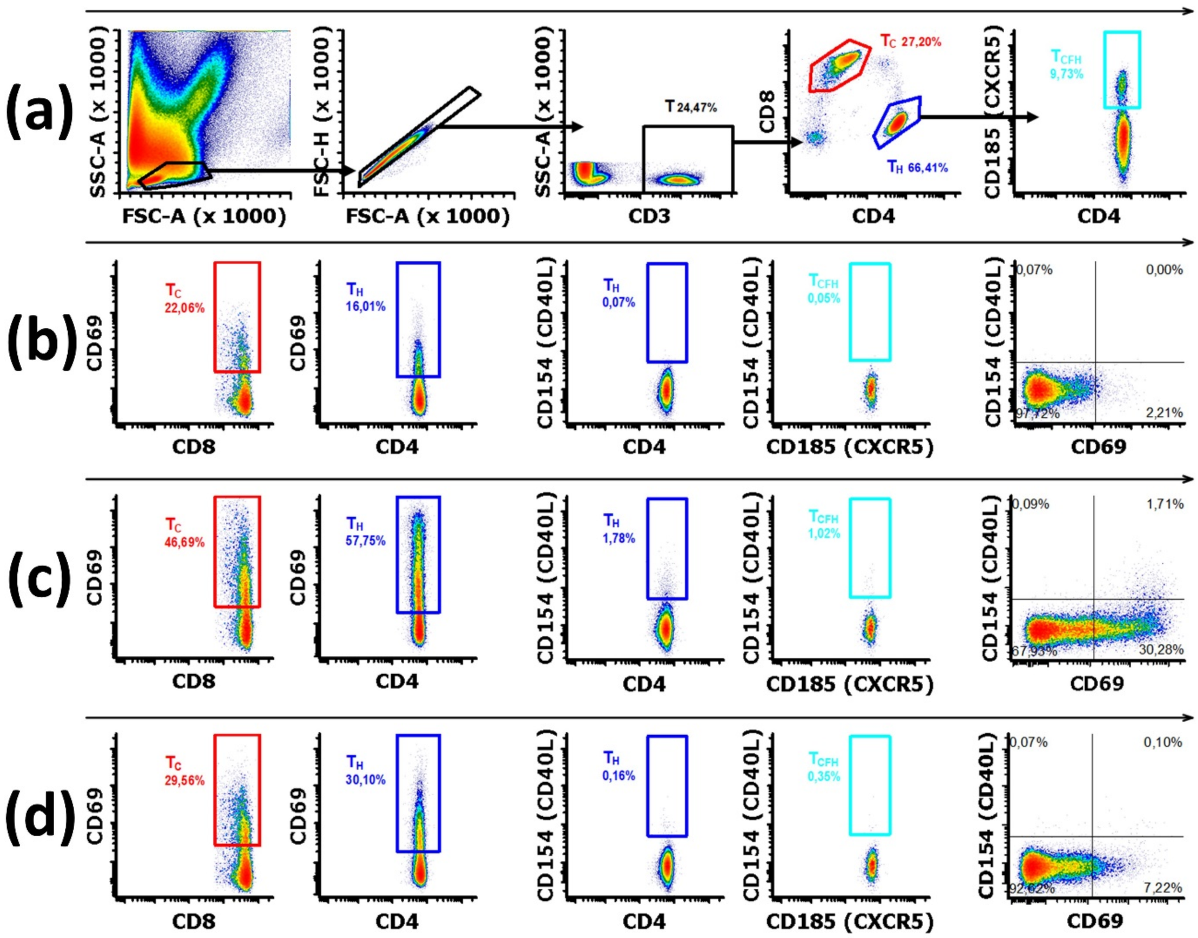

Figure S1. Flowcytometric T cell profiling. (**a**) Gating strategy used to define the different T cell subsets (T_H, T_C and T_{CFH}) and to assess T cell activity using both CD69 and CD40L membrane markers following stimulation of immune cells in whole blood. (**b-d**) A representative example of each test condition from whole blood of a participant at mid-term follow-up. From left to right**:** dot plots showing membrane expression of CD69 within Tc and T_H cells, CD40L expression within T_H and T_{CFH} cells and combined CD69/CD40L expression within T_H in unstimulated (**b**), mitogen stimulated (**c**) and SARS-CoV-2 antigen stimulated cells of a representative study participant (**d**).

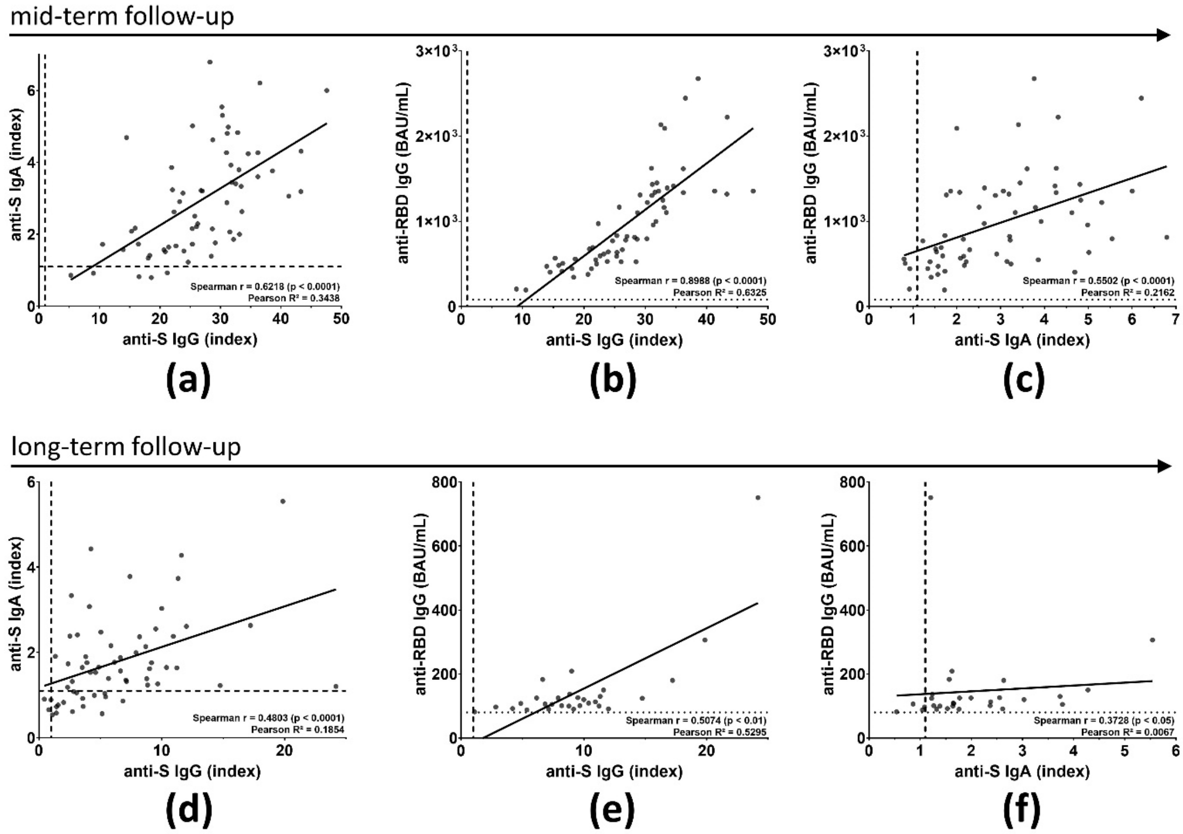

Figure S2. Correlation plots of SARS-CoV-2 specific serology measured at mid- and long-term follow-up. (**a**–**c**) Correlation plots at mid-term follow-up: (**a**) anti-S IgG vs anti-S IgA, (**b**) anti-S IgG vs anti-RBD IgG and (**c**) anti-S IgA vs anti-RBD IgG. Dashed lines = assay-specific cut-offs, dotted lines = assay-specific LOD. (**d**–**f**) Correlation plots at long-term follow-up: (**d**) anti-S IgG vs anti-S IgA, (**e**) anti-S IgG vs anti-RBD IgG and (**f**) anti-S IgA vs anti-RBD IgG. Dashed lines = assay-specific cut-offs, dotted lines = assay-specific LOD. Abbreviations: S = spike, RBD = receptor-binding domain, % IH = percentage inhibition, ACE2 = angiotensin converting enzyme 2, qAC50 = 'qualified AC50': 50 % activity against SARS-CoV-2 variant, VoC = variant of concern, pre = baseline sampling moment before vaccination, 3m = 3 months after baseline, 10m = 10 months after baseline, IQR = interquartile range, LOD = limit of detection.

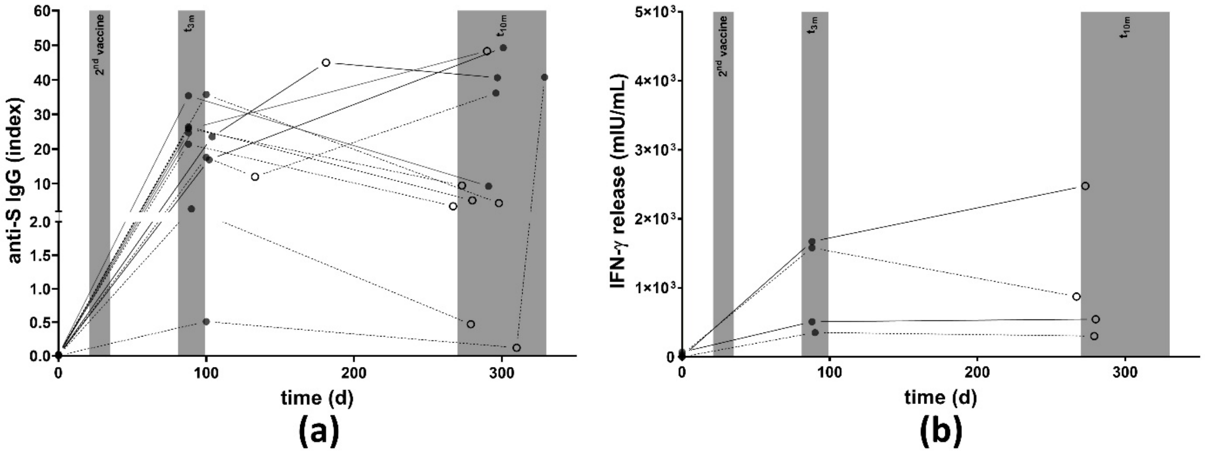

Figure S3. Evolution of SARS-CoV-2 specific humoral and cellular immunity in subjects with reported BTI (n = 11 and 4). (**a**) Anti-S IgG titers. (**b**) T cell mediated IFN-γ release. Open circles titers = t_{BTI} sampling moment (if present). Full lines = subjects with rising anti-S IgG antibodies or specific IFN-γ release at t_{BTI} compared to t_{3m}. Dashed lines = subjects with decreasing anti-S IgG antibodies or specific IFN- γ release at t_{BTI} compared to t_{3m}. Abbreviations: RBD = receptor-binding domain,

IFN-γ = interferon-γ, BTI = breakthrough infection, SD = standard deviation, 3m = 3 months after baseline, 10m = 10 months after baseline.

Table S1. Correlation between RBD specific B cells and humoral parameters measured after BNT162b2 vaccination.

Parameter vs Parameter	t_{3m} Spearman r (*p*-Value)	Pearson R^2	t_{10m} Spearman r (*p*-Value)	Pearson R^2
RBD specific B cells vs anti-S IgG	0.9333 (*p* = 0.0007	0.4425	0.2183 (*p* = 0.4504)	0.0163
RBD specific B cells vs anti-RBD IgG	0.9000 (*p* = 0.002)	0.6326	−0.1094 (*p* = 0.7655)	0.0060
RBD specific B cells vs RBD-ACE2 % IH	0.8833 (*p* = 0.0031)	0.3202	0.2448 (*p* = 0.3961)	0.0495
RBD specific B cells vs qAC50 D614G	0.7333 (*p* = 0.0311)	0.1231	−0.0826 (*p* = 0.7969)	0.0027
RBD specific B cells vs qAC50 delta	0.5988 (*p* = 0.125)	0.0611	0.0952 (*p* = 0.8401)	0.0151

Abbreviations: S = spike, RBD = receptor-binding domain, ACE2 = angiotensin converting enzyme 2, % IH = percentage inhibition, qAC50 = 'qualified AC50': 50 % activity against SARS-CoV-2 variant, 3m = 3 months after baseline, 10m = 10 months after baseline.

Table S2. Demographic and sequencing information of subjects with reported BTI (n = 13).

Subject ID	Age (Years)	Sex (F/M)	t_{BTI} (Days) *	VoC	GISAID ID
074	38	F	44	Alpha	EPI_ISL_7979782
023	34	M	47	Alpha	EPI_ISL_11998002
027	38	F	103	Alpha	TBA
024	26	M	151	Delta	EPI_ISL_5425267
067	34	F	212	Delta	In progress
073	35	F	237	NA	NA
075	36	F	243	NA	NA
061	38	F	249	Delta	TBA
068	37	F	250	Delta	TBA
041	58	M	260	Delta	TBA
052	53	F	264	Delta	TBA
007	45	M	268	Delta	TBA
005	56	M	280	Delta	TBA

* = number of days after receiving the second BNT162b2 vaccine. Abbreviations: ID = coded identity, F = female, M = male, BTI = breakthrough infection, VoC = variant of concern, NA = not available, TBA = to be added.

Table S3. Disease severity and symptoms of subjects with reported BTI (n = 13).

Subject ID	Severity *	Fever	Cough	Sore Throat	Muscle Pain	Malaise	Dyspnea	Sum
074	Mild	1	7	2	1	3	4	18
023	Asymptomatic	1	2	1	1	1	1	7
027	Mild	1	3	1	1	1	1	8
024	Mild	1	2	2	1	4	1	11
067	Asymptomatic	1	1	1	1	1	1	6
073	Mild	4	3	1	1	4	1	14
075	Mild	1	3	1	4	4	2	15
061	Mild	7	1	5	7	7	1	28
068	Mild	4	1	1	1	5	1	13
041	Mild	2	2	1	4	4	1	14
052	Asymptomatic	1	2	1	1	1	1	7
007	Mild	3	2	1	4	6	1	17

005	Mild	4	4	1	3	4	1	17

Symptoms were graded using a scale from 1-10 with 1 = not present and 10 = severe impact on daily life. * = WHO COVID-19 severity score. Abbreviations: ID = coded identity.

DEELNEMER ID: **DATUM:**
Interne referentie AZ Groeninge:
(voorbehouden voor onderzoeker AZ Groeninge)

COV-VAX-AZG	Monitoring ernst van ziekte, herstel en epidemiologie bij deelnemers met positieve COVID test

Beste,

Dank voor uw deelname aan de COV-VAX-AZG studie waarbij de vaccin-geïnduceerde immuunrespons tegen het SARS-CoV-2 virus wordt bestudeerd.
U liet zich eerder testen voor infectie met SARS-CoV-2 met positief testresultaat. Mogen wij a.u.b. vragen om bijkomend onderstaande vragenlijst in te vullen en terug te bezorgen aan [redacted]. Uw gegevens worden geanonimiseerd voor de onderzoekers.

Ziekteverloop	
Koorts	
Hoest	
Pijnlijke keel	
Spierpijn	
Zich algemeen slecht voelen	
Kortademig	
Werd bij u longontsteking vastgesteld?	
**Herstel**	
Duur van ziekte (# dagen)	
Werkverlet (# dagen)	
Ziekenhuisopname (# dagen)	
Opname op intensieve zorgen (# dagen)	
Medicatie (# dagen)	
**Epidemiologie**	
Andere gezinsleden ziek (COVID)?	– indien ja, aantal personen:
Collega's binnen uw dienst ziek (COVID)?	– indien ja, aantal personen:
Werkte u met COVID patiënten binnen AZG?	

DEELNEMER ID: **DATUM:**

WHO severity scoring:
(voorbehouden voor onderzoeker AZ Groeninge)

Methods S1. Template of the in-house developed questionnaire for study participants with breakthrough infection. This questionnaire is in Dutch.
